# Continuous Oral Administration of the Superantigen Staphylococcal Enterotoxin C2 Activates Intestinal Immunity and Modulates the Gut Microbiota in Mice

**DOI:** 10.1002/advs.202405039

**Published:** 2024-09-09

**Authors:** Wu Gu, Huiwen Zhang, Zhichun Zhang, Mingkai Xu, Xiang Li, Zhiyang Han, Xuanhe Fu, Xu Li, Xiujuan Wang, Chenggang Zhang

**Affiliations:** ^1^ Institute of Applied Ecology Chinese Academy of Sciences 72 WenHua Road Shenyang 110016 P. R. China; ^2^ Key Laboratory of Superantigen Research of Liao Ning Province No. 72 WenHua Road Shenyang 110016 P. R. China; ^3^ Department of Immunology Shenyang Medical College No. 146 Huanghe North Street Shenyang 110034 P. R. China; ^4^ Best Health (Guangdong) Bio‐Technology Co., Ltd. Center Building, Minke Park, Xinhui Economic Development Zone Jiangmen 529100 P. R. China; ^5^ University of Chinese Academy of Sciences No.1 Yanqihu East Rd, Huairou District Beijing 101408 P. R. China

**Keywords:** gut microbiota, intestinal barrier, intestinal immune, oral administration, SEC2, superantigen

## Abstract

Staphylococcal Enterotoxin C2 (SEC2), a classical superantigen, is an antitumor immunotherapy agent. However, the injectable formulation of SEC2 limits its clinical application. Here, it is reported that oral administration of SEC2 activates the intestinal immune system and benefits intestinal health in a mouse model. These results indicate that intact SEC2 is detected in the stomach, intestine, and serum after oral administration. Continuous oral administration of SEC2 activates immune cells in gut‐associated lymphoid tissues, promoting extensive differentiation and proliferation of CD4^+^ and CD8^+^ T cells and CD19^+^ B cells, leading to increased production of cytokines and secretory immunoglobulin A. SEC2 also enhances intestinal barrier function, as demonstrated by an increased villus length/crypt depth ratio and elevated expression of mucins and tight junction proteins. Additionally, SEC2 indirectly influenced gut microbiota, reinforcing potential probiotics and short‐chain fatty acid synthesis. Enhanced differentiation of T and B cells in the spleen, coupled with elevated serum interleukin‐2 levels, suggests systemic immune enhancement following oral administration of SEC2. These findings provide a scientific basis for the development of SEC2 as an oral immunostimulant for immune enhancement and anti‐tumor immunotherapy.

## Graphical abstract

Orally administered staphylococcal enterotoxin C2 (SEC2) acts directly on the intestinal mucosal immune system, stimulating the differentiation of T and B cells in the intestine. SEC2 also indirectly alters the structure of the gut microbiota, enhances the production of metabolite short‐chain fatty acid, and improves intestinal barrier function. Thereby, orally administered SEC2 achieves systemic immune enhancement without significant immune‐related side effects.

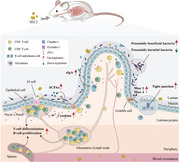

## 1 Introduction

Staphylococcal enterotoxins (SEs) are a kind of superantigen (SAg) produced by gram‐positive *Staphylococcus aureus*.^[^ ^1^ ^]^ Unlike conventional antigens, SEs bind directly to the outside peptide groove of the major histocompatibility complex II (MHC II) on the antigen‐presenting cells (APC) without processing and interact with the specific Vβ region of the T‐cell receptor (TCR).^[^ ^2^ ^]^ This binding pattern enables SEs to effectively stimulate T cells, even at extremely low concentrations, resulting in substantial activation of T lymphocytes and the release of a large quantity of cytokines.^[^ ^3^, ^4^, ^5^ ^]^ Furthermore, SEs can indirectly stimulate B cells by activating T helper 2 (Th2) cells and induce T cell‐dependent B cell differentiation and immunoglobulin production.^[^ ^6^ ^]^ These characteristics make SEs ideal immunomodulators. Staphylococcal Enterotoxin C2 (SEC2), a classic superantigen with effective immunostimulatory activity but relatively low toxicity, has been approved by the State Drug Administration of China (SDA) as an injectable anti‐tumor immunotherapeutic drug for clinical applications since 1999.^[^ ^5^, ^7^ ^]^ Our laboratory is dedicated to the study of SEC2, including modifying its structure for reduced toxicity and increased efficiency,^[^ ^8^, ^9^ ^]^ exploring the molecular mechanisms of T cell activation,^[^ ^7^, ^10^ ^]^ and advancing the development of SEC2 and its mutants as anti‐tumor drugs and vaccine adjuvants.^[^ ^11^, ^12^, ^13^ ^]^ Previous studies on the development of superantigen‐derived drugs have predominantly focused on injectable formulations,^[^ ^5^, ^14^ ^]^ which may limit their future clinical applications. The oral administration route, which has the advantages of easier execution, fewer side effects, and non‐invasiveness, is associated with higher compliance.^[^ ^15^, ^16^ ^]^ Although developing protein drugs for oral administration is challenging, SEC2 is suitable for oral administration because it can tolerate low pH and trypsin to some extent, and can be transcytosed by Caco‐2 cells from the apical to the basolateral surface.^[^ ^17^, ^18^, ^19^ ^]^ Notably, as a bacterial toxin, orally administered SEC2 did not induce obvious gastrointestinal (GI) toxicity in monkey and rat models.^[^ ^17^, ^20^ ^]^ Additionally, it was verified that orally administered SEC2 enhanced the systemic immune function and inhibited the development of transplanted tumors.^[^ ^18^ ^]^ However, the behavior of SEC2 in the gastrointestinal tract (GIT) after oral administration and the precise mechanisms by which orally administered SEC2 activates the intestinal and systemic immune and inhibits tumors remain unclear.

The intestinal mucosal immune system is the primary immune defense system in the human body. Gut‐associated lymphoid tissue (GALT), which houses over 70% of the body's total lymphocytes, plays a vital role in maintaining the systemic immune balance.^[^ ^21^, ^22^ ^]^ Abundant T and B cells within the GALT coordinate the adaptive immune response in the intestine. They produce cytokines and secrete immunoglobulins, which contribute to the modulation of the intestinal immune barrier and the maintenance of mucosal homeostasis.^[^ ^23^, ^24^ ^]^ Furthermore, intestinal immune cells can interact with systemic immunity through circulation in the peripheral blood and lymphatic systems.^[^ ^25^ ^]^ This provides a potential avenue for achieving systemic immune regulation by modulating immune cells in the intestine.

The gut microbiota is a dynamic ecosystem with high diversity and close interactions with homeostasis and the formation of the immune system. Microbiome‐wide association studies have revealed a profound correlation between specific intestinal microbes and the onset of diseases, including tumors, metabolic disorders, and autoimmune conditions.^[^ ^22^ ^]^ Intestinal microbial metabolites, such as short‐chain fatty acids (SCFA), play an important role in mending the intestinal epithelial barrier, mitigating inflammation, and fostering immune cell maturation in GALT.^[^ ^26^, ^27^, ^28^ ^]^ These studied implied that shifts in the diversity of intestinal microbiota and the abundance of certain microbial species are pivotal in influencing both intestinal immunity and the entire immune system. Reciprocally, the immune system is considered one of the most important forces shaping the structure of the gut microbiota through immune‐microbiota crosstalk.^[^ ^29^ ^]^ Innate lymphoid cells are significant regulators of microbial ecology by secreting cytokines such as interleukins, interferon gamma (IFN)‐γ, tumor necrosis factor (TNF)‐α to modulate the gut microbiota composition.^[^ ^30^ ^]^ Increasing evidence has also revealed the crucial role of the adaptive immune system in controlling gut microbiota.^[^ ^31^ ^]^ For instance, B cells, which produce secretory immunoglobulin A (sIgA) that targets specific bacteria, are major contributors to the maintenance of homeostasis within the gut.^[^ ^32^, ^33^ ^]^ These findings indicate that orally administered SEC2 may indirectly affect the gut microbiota. However, there are no reports on the effect of superantigens, especially SEC2, on gut microbiota. Whether and how SEC2 influences the gut microbiota remains to be determined.

In this study, we aimed to comprehensively explore whether a safe dose of long‐term oral SEC2 could activate intestinal immunity without causing intestinal toxicity. Three different doses (high, medium, and low) were selected for continuous oral administration in mice. First, we investigated the distribution of SEC2 and conducted a preliminary safety assessment after its oral administration. Additionally, we analyzed the effect and mechanisms of orally administered SEC2 on intestinal mucosal immunity, gut microbiota, and peripheral immunity. We hope that this study provides theoretical support for the development of SEC2 and its mutants as orally administered immunostimulant drugs to enhance the immune system and for anti‐tumor immunotherapy.

## 2 Result

### 2.1 Orally Administered SEC2 Resides in the Intestinal Tract of Mice

**Figure 1:**
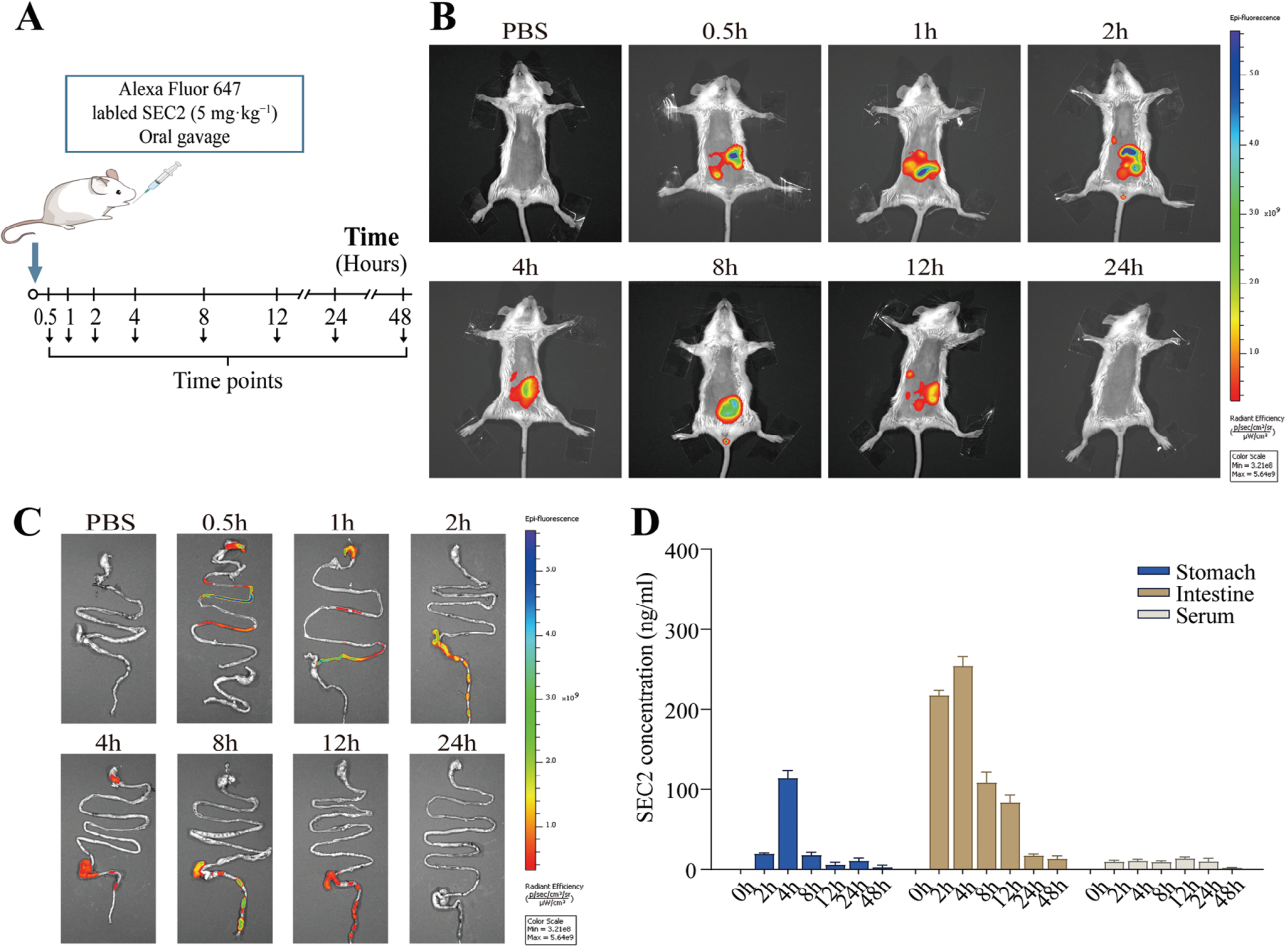
Evaluation of orally administered SEC2 distribution in mice. A) BALB/C mice received intragastric administration of Alexa Fluor 647‐labelled SEC2 and were terminated at each time point. B) The distribution of Alexa Fluor 647‐labelled SEC2 at different time points was determined by near‐infrared fluorescence imaging. C) Fluorescence imaging of isolated GIT. D) SEC2 concentrations in the isolated stomach, intestine, and serum at different time points were determined by sandwich‐ELISA. Data are presented as mean ± SD (n = 3).

To investigate whether orally administered SEC2 could reach and reside in the intestine, intact SEC2 was labeled with Alexa Fluor 647 before intragastric (IG) administration to BALB/C mice (**Figure** **1**A). In vivo imaging results demonstrated that fluorescence signals were detectable in mice from 0.5 to 12 h after administration of labeled SEC2 (Figure 1B). Subsequently, in the digestive tracts of mice dissected at each time point, labeled SEC2 gradually migrated from the stomach to the rectum (Figure 1C). To avoid fluorescent signal interference from degraded labeled proteins and to assess the integrity of residual SEC2 in the GIT, we adopted a Sandwich‐ELISA approach that employs a mouse anti‐SEC2 monoclonal antibody as the capture antibody and exclusively identifies the intact structure of SEC2. The results suggest that intact SEC2 could be detected in both the stomach and intestine from 2 to 48 h, with a peak concentration at 4 h (stomach: 113 ng mL^−1^, intestine: 254 ng mL^−1^). Intact SEC2 was also detected in the serum 2–24 h after oral administration (Figure 1D).

### 2.2 Continuous Oral Administration of SEC2 has no Obvious Toxicity in Mice

**Figure 2:**
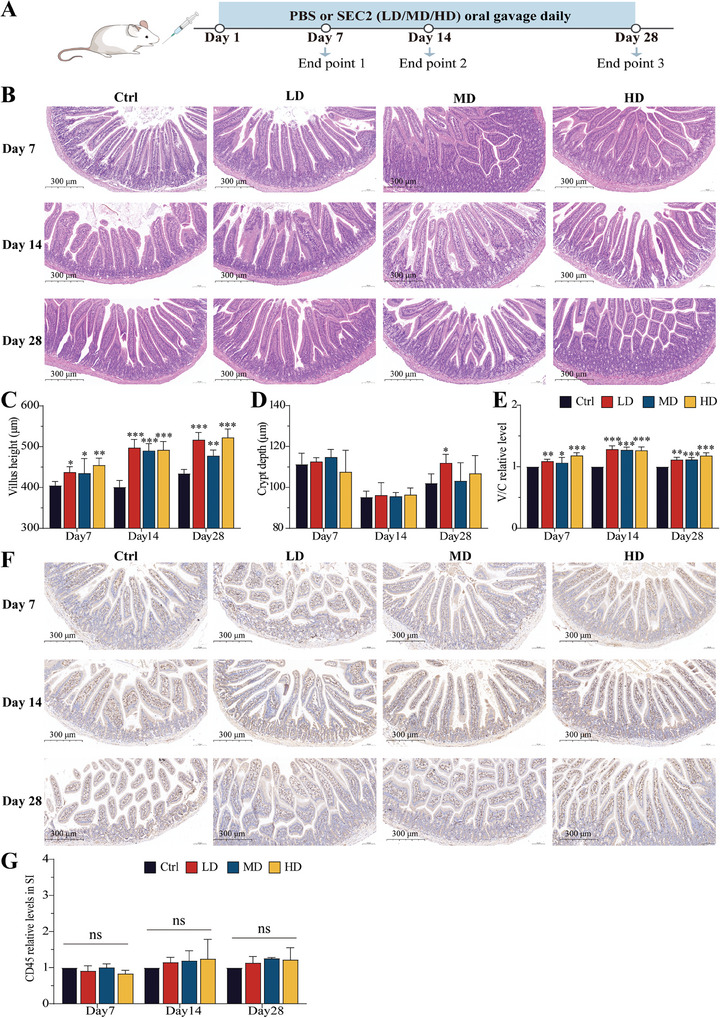
Intestinal morphology and leukocyte (CD45) levels in mice after oral administration of SEC2. A) Schematic overview of the experimental procedure. BALB/C mice were orally administered daily with PBS and SEC2 (at three doses, LD group: 5 mg kg^−1^, MD group: 10 mg kg^−1^, and HD group: 20 mg kg^−1^). The mice were sacrificed at three different time points (Day 7, Day 14, and Day 28). B) Intestinal morphology in mice was evaluated by H&E staining. Scale bar = 300 µm. C) Villus height of each group. D) Crypt depth of each group. E) The ratio of villus height to crypt depth, normalizing the ratio in the treatment groups to those in the control groups. V/C: Villus height/Crypt depth. F) IHC analysis of CD45 for leukocyte infiltration in the small intestine. Scale bar = 300 µm. G) Histograms of the CD45 data were presented through visual analysis. SI: small intestine. Data are presented as mean ± SD (n = 3). “*” represents significant differences between the treatment groups and their respective control groups. *p < 0.05, **p < 0.01, ***p < 0.001. “ns”, not significant.

Mice received SEC2 (LD group: 5 mg kg^−1^, MD group: 10 mg kg^−1^, and HD group: 20 mg kg^−1^) or PBS at a volume of 0.2 mL via oral gavage every day for 7 days, 14 days, or 28 days (**Figure** **2**A). The body weight and defecation of mice in all groups were observed daily during continuous oral administration. The body weight of mice in all groups increased steadily, with the SEC2 and control groups exhibiting similar growth rates, indicating that 28 days of continuous oral administration of SEC2 had no negative effect on the body weights of mice (Figure S1, Supporting Information). Furthermore, no abnormal defecation or other adverse effects were observed in any group.

### 2.3 Continuous Oral Administration of SEC2 Does Not Cause Structural Disruption or Inflammatory Damage to the Intestine

As a bacterial enterotoxin, SEC2 carries the potential risk of inducing diarrhea and causing intestinal inflammatory damage. Hematoxylin and eosin (H&E) staining and histological scoring were used to assess the effect of SEC2 on the histopathology and morphology of the small intestine in mice after oral administration. As shown in Figure 2B and Figure S2 (Supporting Information), the small intestinal villi of mice in all the SEC2 groups were structurally intact and densely arranged, with well‐developed crypts, and no pathological features of villous edema or epithelial damage were observed. Visualization analysis revealed that continuous oral administration of SEC2 increased the length of the villi and resulted in an elevated villus height to crypt depth (V/C) ratio, which was most significant after 14 days of continuous administration (Figure 2C–E) (*p* < 0.001). These results indicated that continuous oral administration of SEC2 did not cause structural disruption of the small intestine.

CD45 is a leukocyte common antigen and immunohistochemical analysis of CD45 reflects the extent of inflammatory cell infiltration into tissues. As shown in Figure 2F,G, the level of CD45‐positive cells in the small intestinal tissues of mice in all the SEC2 groups did not show any significant change during the 28 days of continuous oral administration, suggesting that long‐term continuous oral administration of SEC2 did not cause inflammatory damage in the intestine.

### 2.4 Continuous Oral Administration of SEC2 Activates T and B Cells in Peyer's Patches and Mesenteric Lymph Nodes

Peyer's patches (PPs), located on the inner side of the small intestinal mucosa, and mesenteric lymph nodes (MLNs), distributed along the mesenteric arteries on the outer side of the intestine, serve as the primary sites of intestinal mucosal immunity, containing a significant population of immune cells. To validate the activation effect of orally administered SEC2 on intestinal mucosal immune cells, flow cytometry was used to analyze the differentiation of T and B cells in PPs and MLNs.

**Figure 3:**
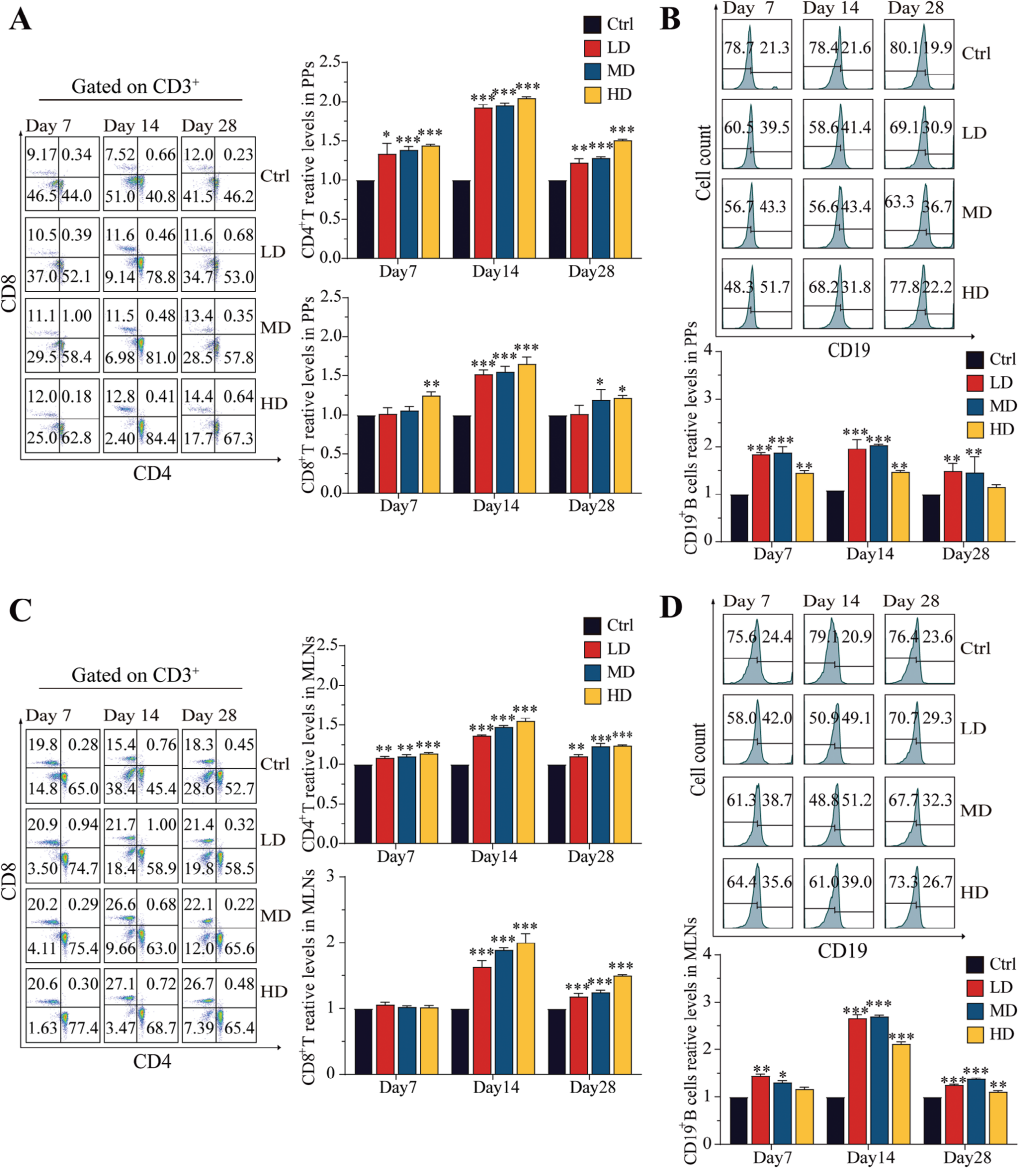
Stimulation of T‐ and B‐lymphocyte differentiation and proliferation in the mouse PPs and MLNs after oral administration of SEC2. A) Flow cytometry results of T‐lymphocyte differentiation in PPs after oral administration of SEC2. The proportion of CD4^+^ and CD8^+^ T lymphocytes were gated on CD3^+^ cells. B) Flow cytometry results of B‐lymphocyte proliferation in PPs after oral administration of SEC2. C) Flow cytometry results of T‐lymphocyte differentiation in MLNs after oral administration of SEC2. The proportion of CD4^+^ and CD8^+^ T lymphocytes were gated on CD3^+^ cells. D) Flow cytometry results of B‐lymphocyte proliferation in MLNs after oral administration of SEC2. Data were normalized with the control group set as 1 and presented as mean ± SD (n = 3). “*” represents significant differences between the treatment groups and their respective control groups. *p < 0.05, **p < 0.01, ***p < 0.001.

As shown in **Figure** **3**A, in PPs, the proportions of both CD4^+^ and CD8^+^ T cells significantly increased following continuous oral administration of SEC2, peaking on day 14. Additionally, the ratios of both CD4^+^ and CD8^+^ T cells increased with increasing doses of SEC2, demonstrating a certain dose‐dependency in the activation of T cells. Notably, activation of the CD4^+^ T cell subset by SEC2 was more pronounced than that of the CD8^+^ T cell subset. The proliferation of B cells in PPs is shown in Figure 3B. Continuous oral administration of SEC2 significantly increased the proportion of CD19^+^ B cells, peaking on day 14. In contrast to the dose dependency observed in SEC2‐induced T cell activation, the activation of B cells by SEC2 were attenuated in HD groups. As shown in Figure 3C,D, continuous oral administration of SEC2 significantly increased the proportions of CD4^+^ T cells, CD8^+^ T cells, and CD19^+^ B cells in the MLNs of mice. The activating effect of SEC2 on T and B cells in the MLNs followed a trend similar to that observed in the PPs.

### 2.5 Continuous Oral Administration of SEC2 Induces the Transcription of Cytokines in the Intestine

**Figure 4:**
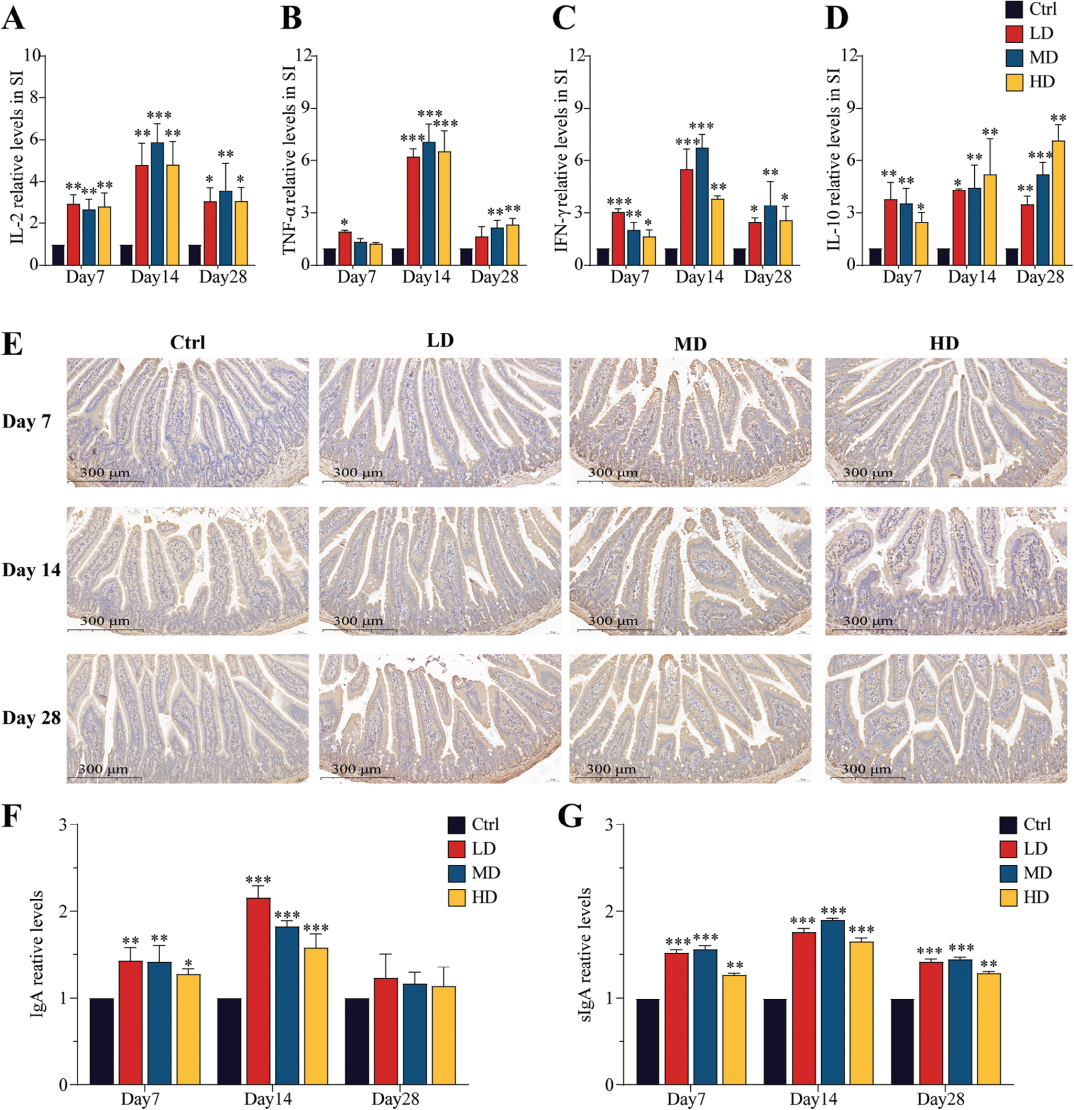
Stimulation of cytokine and sIgA expression in the mouse small intestine after oral administration of SEC2. A–D) The relative transcription levels of IL‐2, TNF‐α, IFN‐γ, and IL‐10 genes in the mouse intestine. SI: small intestine. E) IgA cell proliferation in the small intestine was analyzed by IHC. Scale bar = 300 µm. F) Histograms of the IgA data were presented through visual analysis. G) ELISA results for sIgA. Data were normalized with the control group set as 1 and presented as mean ± SD (n = 3). “*” represents significant differences between the treatment groups and their respective control groups. *p < 0.05, **p < 0.01, ***p < 0.001.

Cytokine transcription levels in the small intestine were assessed by RT‐qPCR. As shown in **Figure** **4**A–D, the transcription levels of both pro‐inflammatory cytokines (IL‐2, TNF‐α, IFN‐γ) and the anti‐inflammatory cytokine (IL‐10) were increased in all SEC2 groups when compared to the Ctrl group. Notably, IL‐2, TNF‐α, and IFN‐γ displayed similar upregulation patterns, with each group reaching its peak at day 14. It was noteworthy that the MD group exhibited a higher upregulation ratio than the LD and HD groups. After 28 days of continuous administration, the transcription levels of these pro‐inflammatory cytokines decreased but remained significantly higher than those in the control group (Figure 4A–C). In contrast, the transcription levels of the anti‐inflammatory cytokine IL‐10 demonstrated a consistent upward trend with prolonged administration, exhibiting a positive correlation with the escalating dose of SEC2 (Figure 4D). This trend aligned with the attenuated upregulation patterns of pro‐inflammatory cytokines observed in the HD and day 28 groups.

### 2.6 Continuous Oral Administration of SEC2 Increases the IgA and the sIgA

Secretion of sIgA into the intestinal lumen by IgA^+^ plasma cells in the lamina propria. It plays a crucial role in intestinal mucosal immunity, serving as the primary defense in preserving intestinal microbiota homeostasis and protecting the gut from pathogenic invasion.

An immunohistochemical (IHC) assay was performed to detect the distribution of IgA in the lamina propria of the small intestine, and the results are presented in Figure 4E,F. A significant increase in IgA content was observed on days 7 and 14 in all of the SEC2 groups. The increase in IgA reached its peak on day 14 but was negatively correlated with the dose of SEC2. On day 28, no significant difference in IgA levels was observed compared to the control group. sIgA levels in the small intestine were measured using ELISA (Figure 4G). Compared with the control group, sIgA levels were significantly upregulated in all SEC2 groups at all three time points (*p* < 0.01). Consistent with the IHC detection of IgA, the expression of sIgA increased with the duration of continuous administration and peaked on day 14. However, the MD group exhibited the most substantial upregulation in sIgA expression (*p* < 0.001).

### 2.7 Continuous Oral Administration of SEC2 Activates the Peripheral Immune System

**Figure 5:**
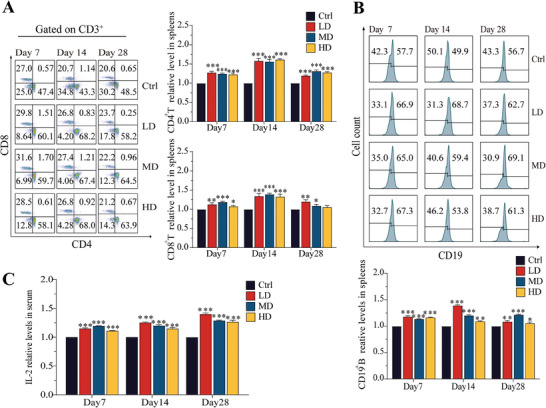
Activation of the mouse peripheral immune system after oral administration of SEC2. A) Flow cytometry results of T‐lymphocyte differentiation in spleens. The proportion of CD4^+^ and CD8^+^ T lymphocytes were gated on CD3^+^ cells. B) Flow cytometry results of B‐lymphocyte proliferation in spleens. C) ELISA results for IL‐2 in serum. Data were normalized with the control group set as 1 and presented as mean ± SD (n = 3). “*” represents significant differences between the treatment groups and their respective control groups. *p < 0.05, **p < 0.01, ***p < 0.001.

Continuous oral administration of SEC2 led to a substantial increase in the differentiation of both CD4^+^ and CD8^+^ T cells in the spleen (*p* < 0.001 and *p* < 0.05, respectively), with the peak reaching day 14, but without a dose‐dependent pattern (**Figure** **5**A). The differentiation of CD19^+^ B cells in the spleen also significantly increased in all of the SEC2‐treated groups, albeit to a lesser magnitude than that of T cells. However, there was no obvious dose‐ or time‐dependent change in CD19^+^ B cell differentiation (Figure 5B). Serum IL‐2 levels in all SEC2 groups were significantly elevated compared to those in the control group (*p* < 0.001) and showed a consistent increase with the prolonged duration of administration (Figure 5C). However, at the same time points, no clear correlation was observed between IL‐2 levels and dosage. These results indicated that despite only trace amounts of SEC2 being detected in the serum, oral administration of SEC2 effectively activated the peripheral immune system.

### 2.8 Continuous Oral Administration of SEC2 Promotes Intestinal Barrier Function

Mucins and tight junction proteins within the intestinal epithelium are important for preserving intestinal barrier integrity.

**Figure 6:**
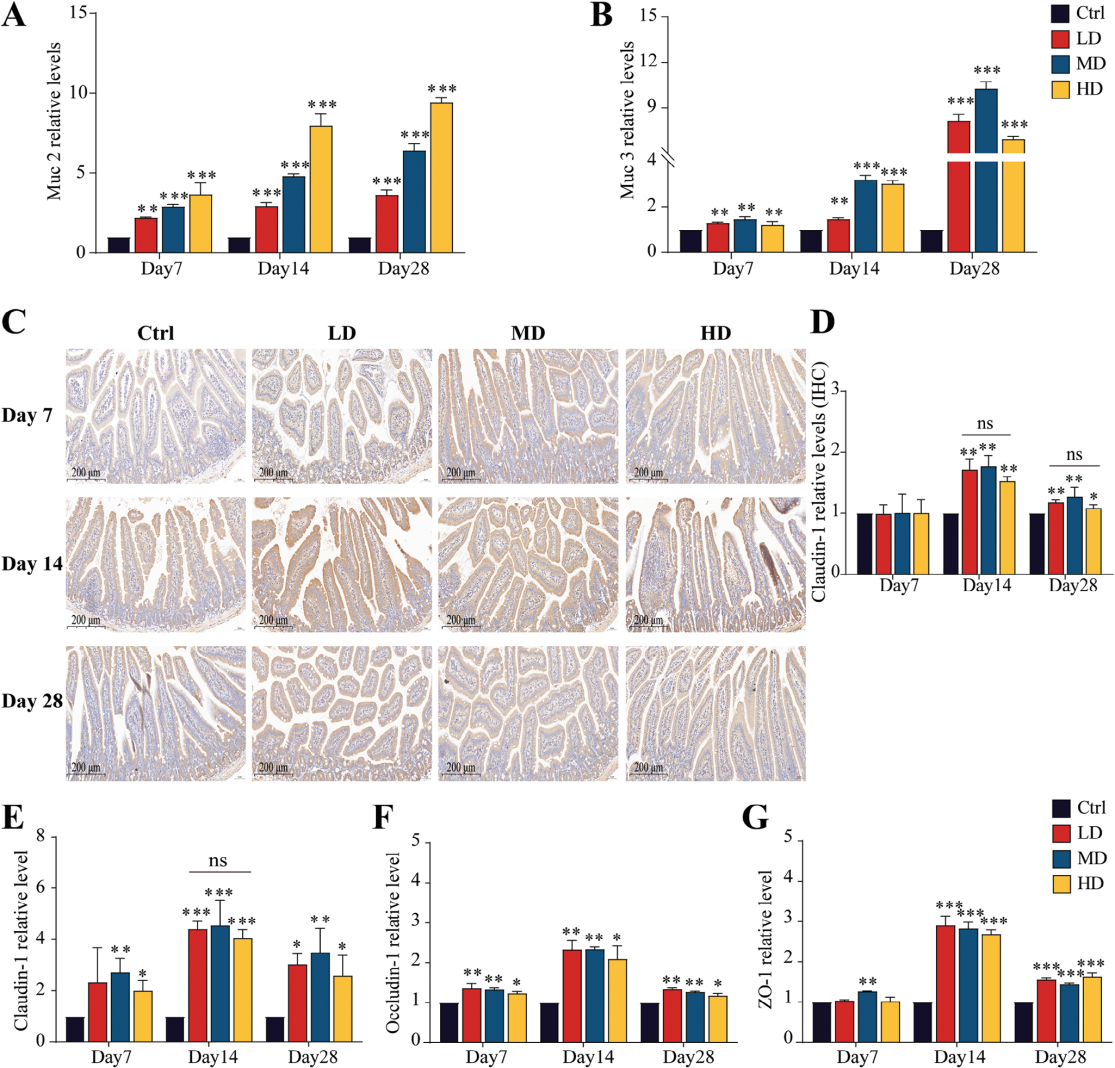
Promotion of intestinal mucins and tight junction proteins after oral administration of SEC2. A,B) The relative transcription levels of Muc2 and Muc3 genes in the mouse intestine. C) Intestinal Claudin‐1 protein expression was analyzed by IHC. Scale bar = 200 µm. D) Histograms of the Claudin‐1 data were presented through visual analysis. E–G) The relative transcription levels of Claudin‐1, Occludin‐1, and ZO‐1 genes in the mouse intestine. Data were normalized with the control group set as 1 and presented as mean ± SD (n = 3). “*” represents significant differences between the treatment groups and their respective control groups. *p < 0.05, **p < 0.01, ***p < 0.001. “ns”, not significant.

Mucins are primarily secreted by goblet cells. Periodic acid‐Schiff (PAS) staining was used to identify the changes in the number of goblet cells in the mouse small intestine. As shown in Figure S3 (Supporting Information), compared to the control group, the number of goblet cells in the villi and crypts of mice orally administered SEC2 was significantly upregulated. This upregulation demonstrated an increasing trend with the treatment duration. Although the upregulation of goblet cells in the villi was slightly reduced in the Day 28‐HD group, it remained significantly elevated in the crypts, indicating an overall increasing trend in goblet cell numbers in the small intestine with prolonged drug administration. This suggests that the continuous oral administration of SEC2 stimulates the sustained production of goblet cells, which predicts enhanced mucin secretion. Muc2 and Muc3 are the predominant mucins in the intestine. Muc2 functions as a secretory mucin and Muc3 serves as a transmembrane mucin. The transcript levels of the mucins Muc2 and Muc3 in the small intestinal epithelium are shown in **Figure** **6**A,B. After continuous oral administration of SEC2, the levels of Muc2 and Muc3 transcription in the mouse intestine significantly increased with prolonged administration (*p* < 0.01), reaching their maximum levels on day 28. As the duration of continuous administration increased, Muc2 displayed a dose‐dependent pattern, whereas Muc3 exhibited the most substantial upregulation in the MD groups.

The primary tight junction proteins in the mouse intestine were analyzed. Claudins and Occludins are transmembrane proteins. Claudin‐1 is vital for the formation of tight junctions, while Occludin‐1 is crucial for maintaining structural integrity. Zonula Occludens (ZO) is a peripheral membrane protein, and ZO‐1 is capable of interacting with intracellular Occludin to establish stable tight junctions. The results of IHC detection revealed that continuous oral administration of SEC2 for 14 and 28 days resulted in a significant upregulation of Claudin‐1 in the intestinal epithelial cells compared to that in the control group (*p* < 0.05); the most pronounced upregulation occurred on day 14, with no substantial difference noted among the three doses (Figure 6C,D). The transcription levels of the tight junction proteins Claudin‐1, Occludin‐1, and ZO‐1 were analyzed using RT‐qPCR. As shown in Figure 6E, the transcription levels of Claudin‐1 exhibited an upregulation trend, which was consistent with the IHC results. The most notable upregulation of Claudin‐1 occurred on day 14, without substantial variation among the different doses. Occludin‐1 and ZO‐1 also showed a noticeable upregulation pattern on days 14 and 28, with the most notable upregulation observed on day 14 (Figure 6F,G). These results suggest that the continuous oral administration of SEC2 promotes intestinal barrier function.

### 2.9 Continuous Oral Administration of SEC2 Alters the Composition of Gut Microbiota

Although SEC2 is produced by *Staphylococcus aureus*, there are currently no reports on the presence of the SEC2 receptor in microorganisms, and there is no evidence that SEC2 can directly interact with microorganisms. Nevertheless, considering the complex interplay between the intestinal mucosal immune system and gut microbiota, we hypothesized that oral administration of SEC2 could indirectly influence the composition of the gut microbiota by modifying intestinal immunity. To investigate whether and how oral administration of SEC2 influences the composition or abundance of the gut microbiota, we analyzed mouse gut microbiota using 16S rRNA high‐throughput sequencing technology.

**Figure 7:**
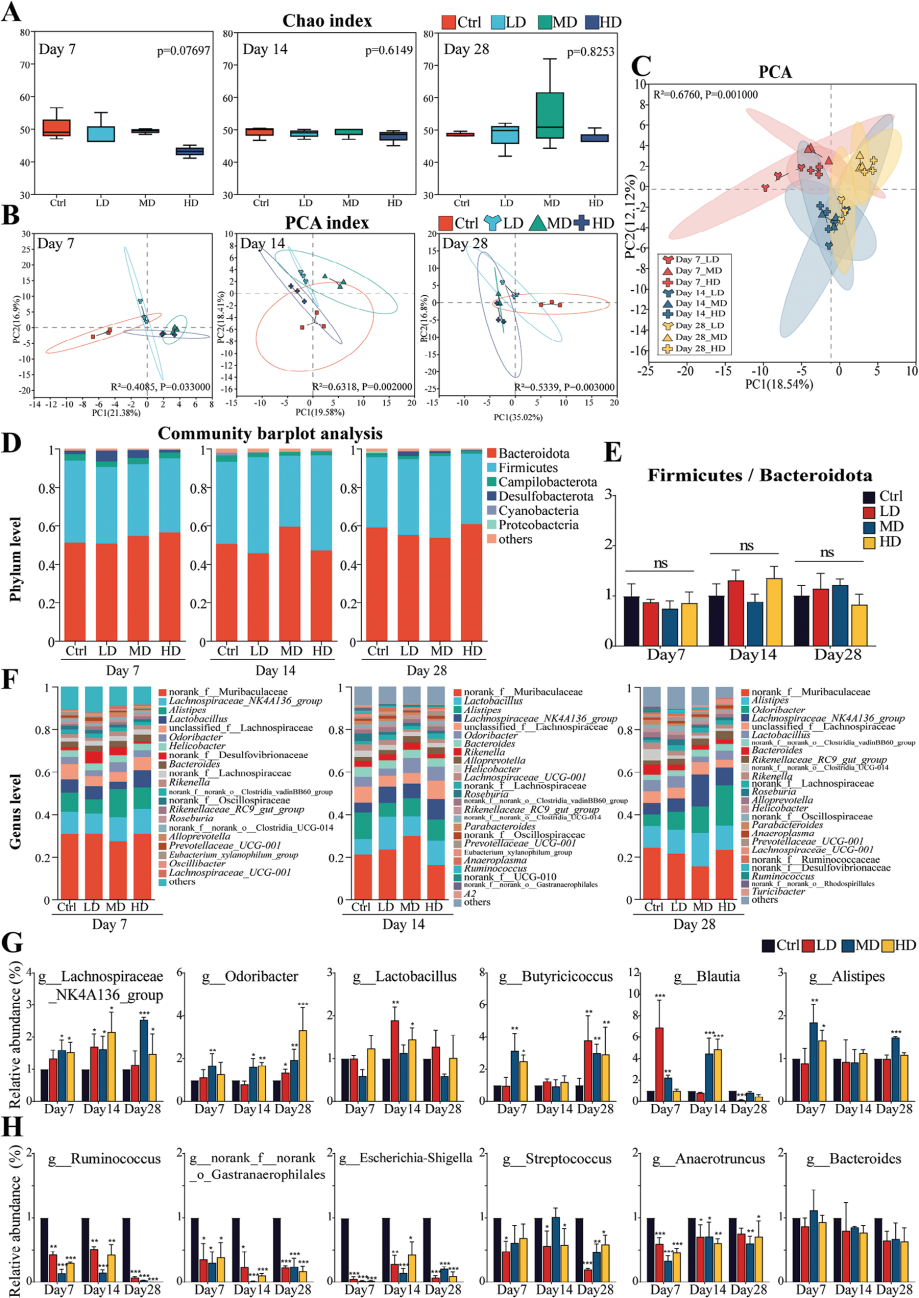
16S rRNA sequencing analysis of alterations in the gut microbiota of mice after oral administration of SEC2. A) Alpha diversity base on Chao index, *p* values are obtained by Kruskal‐Wallis H test. B,C) Beta diversity indices determined by principal component analysis (PCA) of different groups for 7, 14, and 28 days administration and different time points together. D) Phylum‐level composition of gut microbiota. E) Relative abundance of Firmicutes‐to‐Bacteroidetes ratio. F) Genus‐level composition of gut microbiota. G,H) Relative changes in the key genera in response to oral administration of SEC2. Data were normalized with the control group set as 1 and presented as mean ± SD (n = 3). G) upregulated genera, including *Lachnospiraceae_NK4A136_group*, *Odoribacter*, *Lactobacillus*, *Butyricicoccus*, *Blautia*, and *Alistipes*. H) downregulated genera, including *Ruminococcus*, norank_f_norank_o_*Gastranaerophilales*, *Escherichia‐Shigella*, *Streptococcus*, *Anaerotruncus*, and *Bacteroides*. “*” represents significant differences between the treatment groups and their respective control groups. *p < 0.05, **p < 0.01, ***p < 0.001.

Analysis of alpha diversity based on the Chao index showed no significant difference in the community richness of the gut microbiota after continuous oral administration of SEC2 compared to that in the control group (**Figure** **7**A). However, the principal component analysis (PCA) score plot revealed noteworthy distinctions between the SEC2 and control groups at each time point, with these disparities intensifying on days 14 and 28 (*p* = 0.002 and *p* = 0.003, respectively) (Figure 7B). Furthermore, the PCA results for all the SEC2 groups indicated clear clustering patterns among the groups with different dose levels at the same time point (Figure 7C). Continuous oral administration of SEC2 has the potential to modify the structural composition of the mouse intestinal microbiota, and the duration of administration, rather than the dose, exerted a more substantial impact on the gut microbiota.

At the phylum level, Firmicutes and Bacteroidetes were the dominant phyla in all groups, constituting over 90% of the total abundance (Figure 7D). No statistically significant differences were observed in the Firmicutes‐to‐Bacteroidetes (F/B) ratios among the groups at any time point (Figure 7E). Furthermore, at the genus level, the microbial composition in the SEC2 group exhibited distinct bacterial profiles compared with those in the control group (Figure 7F). Differentially abundant genera in response to the SEC2 treatment were further identified through LEfSe analysis (Figures S4 and S5, Supporting Information), and the operational taxonomic units (OTUs) corresponding to these taxa were subjected to statistical analyses to evaluate their relative changes. Six core bacterial genera or potential beneficial genera including *Lachnospiraceae_NK4A136_group* were enriched (Figure 7G), while six potentially harmful genera including *Escherichia‐Shigella* were depleted by SEC2 treatment (Figure 7H). Moreover, both the enrichment of beneficial bacteria (*Lachnospiraceae_NK4A136_group*, *Lactobacillus*, *Odoribacter* etc.) and the depletion of potentially pathogenic bacteria (*Ruminococcus*, *norank_f_norank_o_Gastranaerophilales*, *Escherichia‐Shigella*, *Streptococcus*, etc.) demonstrated an enhanced trend with the extension of the administration period. These results indicate that the continuous oral administration of SEC2 leads to alterations in the composition and structure of the gut microbiota, which may be beneficial for intestinal health.

### 2.10 Alteration of SCFAs in Feces

**Figure 8:**
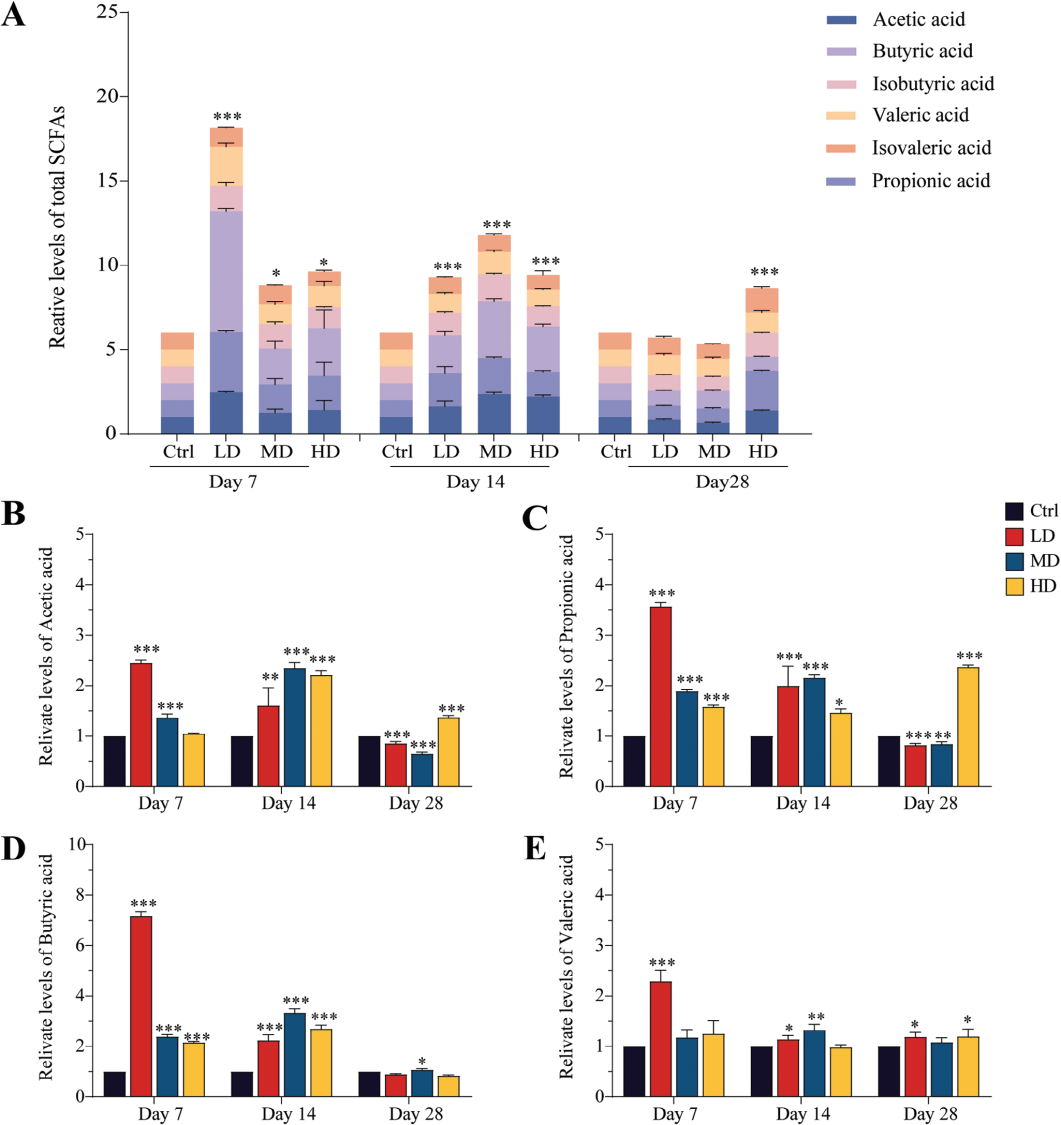
Changes in SCFAs after oral administration of SEC2. A) Total SCFAs in feces. B–E) Relative levels of four major SCFAs: acetic acid, propionic acid, butyric acid, and valeric acid. Data were normalized with the control group set as 1 and presented as mean ± SD (n = 3). “*” represents significant differences between the treatment groups and their respective control groups. *p < 0.05, **p < 0.01, ***p < 0.001.

SCFAs are mainly produced by the intestinal microbiota. SCFAs provide nutrients and energy to intestinal epithelial cells to maintain the integrity and function of the intestinal mucosal barrier and play a pivotal role in preserving the equilibrium between the gut microbiota and the mucosal immune system. In this study, GC‐MS was used to measure the concentrations of six major SCFAs (acetic acid, isobutyric acid, propionic acid, butyric acid, isovaleric acid, and valeric acid) in mouse feces. Standard curves and correlation coefficients (R^2^) are shown in Figure S6 (Supporting Information). The total ion current chromatograms of the SCFAs are listed in Table S1 (Supporting Information). As depicted in **Figure** **8**A, after the oral administration of SEC2, the total SCFAs content increased, particularly exhibiting a significant increase on day7 and day 14 in all SEC2 groups. However, on day 28, SCFA levels in the LD and MD groups gradually reverted to no significant difference compared to those in the control group, whereas SCFA levels in the HD group remained significantly higher than those in the control group. Major variations were observed in the levels of acetic acid, propionic acid, butyric acid, and valeric acid (Figure 8B–E). Of these, butyric acid displayed the most pronounced response to SEC2 administration and was highly upregulated in all SEC2 groups on days 7 and 14 (*p* < 0.001). Alterations in SCFA correlated with both the duration and dose of SEC2 administration. The most substantial upregulation of SCFA occurs with short duration and low dosage, while prolonged administration at high dosage maintains a sustained increase in SCFA levels.

## 3 Discussion

The superantigen SEC2 has been extensively investigated in our laboratory for structural modifications aimed at enhancing its efficacy and attenuating its toxicity. Moreover, we explored its potential application as a novel drug for immune enhancement and anti‐tumor immunotherapy.^[^ ^11^, ^12^, ^13^ ^]^ Therefore, the development of an oral SEC2 formulation would greatly facilitate its clinical application. The oral administration of SEC2 inhibits transplanted tumors in animal models;^[^ ^17^ ^]^ however, the underlying mechanism remains unclear. Furthermore, given that SEC2 exhibits a certain tolerance to the GI environment and considering the substantial influence of intestinal mucosal immunity on systemic immunity, we hypothesized that oral administration of SEC2 may indirectly affect systemic immunity by modulating intestinal immunity.

There are inevitable challenges associated with the oral administration of protein drugs, including exposure to the acidic environment, enzymes, mucosal proteins, and gut microbiota in the GIT.^[^ ^34^ ^]^ In the present study, we verified that SEC2 could be retained as an intact protein in the mouse intestine for up to 12 h after oral administration. Additionally, intact SEC2 was detected in serum, indicating that orally administered SEC2 could be absorbed and transferred by the digestive tract into the bloodstream.

As crucial components of GALT, PPs and MLNs contain a substantial number of immune cells. They serve as primary sites for antigen uptake in the intestinal tract and play a vital role in modulating the intestinal immune barrier.^[^ ^35^, ^36^ ^]^ When foreign antigens are taken up by PPs, activated lymphocytes migrate to the MLNs for further proliferation and activation. Subsequently, activated immune cells return to the intestinal mucosal lamina propria through a cyclic homing process and further differentiate into IgA‐secreting plasma cells and effector T cells, which are the “main forces” in maintaining intestinal mucosal immunity.^[^ ^37^ ^]^ In this study, after the continuous oral administration of SEC2, noticeable T cell differentiation and B cell proliferation were observed in both PPs and MLNs. The proportions of CD4^+^ and CD8^+^ T cells, and CD19^+^ B cells in SEC2‐administered groups increased significantly with the duration of administration. Differentiated CD4^+^ T‐lymphocytes migrate to the intestinal lamina propria to produce various cytokines, thereby exerting cellular immune functions.^[^ ^25^ ^]^ Under the influence of cytokines, CD8^+^ T lymphocytes differentiate into cytotoxic T lymphocytes (CTLs) that directly kill tumor cells. This is the primary mechanism underlying the immune anti‐tumor effects of superantigen.^[^ ^13^ ^]^ RT‐qPCR results revealed that the transcription of cytokines IL‐2, TNF‐α, IFN‐γ, and IL‐10 were significantly upregulated by orally administered SEC2 at all concentrations and time points. T helper 1 (Th1)‐type cytokines IL‐2, TNF‐α, and IFN‐γ are pivotal in cell‐mediated antimicrobial and antiviral immune responses against intestinal pathogens.^[^ ^24^ ^]^ TNF‐α and IFN‐γ can promote plasma cells to secret sIgA into the intestinal lumen.^[^ ^38^ ^]^ Th2‐type cytokine IL‐10 stimulates the maturation and differentiation of B cells into IgA^+^ plasma cells.^[^ ^39^, ^40^ ^]^ Moreover, as an important anti‐inflammatory factor, IL‐10 inhibits the overexpression of inflammatory factors and plays a key role in maintaining the balance of the intestinal immune system. As primarily produced by Th1 cells, in this study, the transcriptions of IL‐2, TNF‐α, and IFN‐γ exhibited a similar pattern as CD4^+^ T cell differentiation. The peaks occurred on day 14 in the MD groups and then decreased with higher dosage or prolonged administration, but remained significantly higher than in the control group. Contrary to the three Th1‐type cytokines, the transcription of IL‐10 continued to increase with increasing SEC2 dosage and prolonged administration and reached a peak at day 28 in the HD group. The sustained elevation of IL‐10 might suppress the amplification of the inflammatory response, which elucidates the downtrend of IL‐2, TNF‐α, and IFN‐γ observed at day 28 in HD groups. Furthermore, H&E staining and CD45 immunohistochemistry demonstrated that the intestinal tissue remained intact and no inflammatory damage to the intestinal epithelial cells was observed in any of the SEC2‐treated groups, even on day 28 in the HD groups. These results suggest that orally administered SEC2 initiates an intestinal immune response by upregulating Th1 cytokines. Additionally, the heightened Th2 anti‐inflammatory factor IL‐10 regulates a sustained increase in inflammatory factors, contributing to the maintenance of intestinal immune homeostasis.

sIgA, synthesized and secreted by IgA^+^ plasma cells in the intestinal lamina propria, is the most abundant subtype of defense antibodies in the human GIT.^[^ ^41^ ^]^ The sIgA can bind and agglutinate intestinal pathogenic bacteria, thereby hindering bacterial attachment and invasion of host intestinal epithelial cells, making it a critical indicator of intestinal mucosal immunity.^[^ ^42^, ^43^ ^]^ After continuous oral administration of SEC2, there was a significant increase in the number of IgA^+^ cells in the lamina propria of the mouse intestinal tract, along with elevated sIgA secretion into the intestinal lumen. This pattern was consistent with the proliferation of CD19^+^ B lymphocytes in the PPs and MLNs. Given that there are currently no reports on the presence of SEC2 receptors on the surface of B cells,^[^ ^13^ ^]^ we speculate that orally administered SEC2 stimulates cytokine production by CD4^+^ T lymphocytes in the intestinal tract, indirectly enhancing the differentiation of B lymphocytes and the maturation of IgA plasma cells. This cascade results in abundant sIgA expression, contributing to the humoral immune function in the intestine.

The spleen is the largest peripheral immune organ in mammals, and serves as a crucial site for immune cell homing.^[^ ^44^ ^]^ Continuous oral administration of SEC2 not only increased the levels of the cytokine IL‐2 in mouse serum, but also significantly upregulated CD4^+^ T, CD8^+^ T, and CD19^+^ B lymphocytes in the spleen, consistent with the observed alterations in GALT. We hypothesized that the oral administration of SEC2 affects peripheral immunity through the following mechanisms. First, since SEC2 was detected in the serum, it might be absorbed into the bloodstream through the intestine and then migrate to the spleen, where SEC2 activates lymphocytes. Second, when SEC2 is absorbed into the bloodstream through the intestinal tract, it leads to the abundant production of IL‐2 in the blood, collectively activating T and B lymphocytes in the bloodstream. Subsequently, these activated lymphocytes migrate to the spleen through bloodstream circulation. Third, T and B lymphocytes in PPs and MLNs are stimulated to proliferate and differentiate by SEC2 and then migrate directly to the spleen through bloodstream circulation. In brief, oral administration of SEC2 can activate peripheral immunity, which partially explains the mechanism of inhibition of transplanted tumors by orally administered SEC2.

The integrity of the intestinal barrier depends on intact epithelial cells, mucosal proteins, and tight junction proteins.^[^ ^45^ ^]^ Although traditional theories suggest that SEC2 is an enterotoxin capable of causing diarrhea, vomiting, and intestinal inflammation, this study found that orally administered SEC2 did not cause any damage to the intestinal epithelium, villi, crypts, or the body weight of adult mice. Additionally, after continuous oral administration of SEC2, there was a significant increase in the V/C ratio, number of goblet cells, and mucin expression levels in the mouse intestine, all of which showed a similar trend of upregulation. These findings indicate that SEC2 promotes the proliferation of goblet cells and the dynamic migration of intestinal epithelial cells and reinforces the chemical barrier maintained by intestinal epithelial cells. This also provides a foundation for the enrichment of functional microbiota in the intestine.^[^ ^46^, ^47^ ^]^ Moreover, the tight junction proteins serve as core components that physically hamper microbial invasion via the paracellular pathway, thereby maintaining the integrity of the intestinal epithelial barrier.^[^ ^48^ ^]^ The significant upregulation of Claudin‐1, Occludin‐1, and ZO‐1 expression suggests that oral administration of SEC2 can enhance the physical barrier function of the intestine. Elevated levels of mucin and tight junction proteins protect the intestine from inflammatory damage.

Intestinal mucosal immune function is regulated by the gut microbiota, and intestinal immune cells and their secreted immune factors can influence the composition of the gut microbiota.^[^ ^49^ ^]^ In this study, we found that continuous oral administration of SEC2 significantly influenced the structural composition of the mouse intestinal microbiota, but did not alter the richness of the community. At the phylum level, a drastic change in the F/B ratio is regarded as dysbiosis.^[^ ^50^ ^]^ The lack of a significant difference in the F/B ratio observed in this study indicates that long‐term oral administration of SEC2 does not result in dysbiosis of the gut microbiota. Analysis of the abundance of individual bacteria revealed alterations in gut microbiota following oral administration of SEC2. Potentially beneficial bacteria, including *Lachnospiraceae_NK4A136_group*, *Odoribacter*, *Lactobacillus*, *Butyricicoccus*, *Blautia*, and *Alistipes*, were widely upregulated. These bacteria are associated with SCFAs production, enhancement of intestinal barrier function, and suppression of inflammation and tumor development.^[^ ^51^, ^52^, ^53^, ^54^, ^55^, ^56^, ^57^, ^58^ ^]^ Conversely, potentially harmful bacteria linked to inflammation, epithelial barrier damage, and the onset of intestinal diseases exhibited a widespread downregulation, including *Ruminococcus*, norank_f_norank_o_*Gastranaerophilales*, *Escherichia‐Shigella*, *Streptococcus*, *Anaerotruncus*, and *Bacteroides*.^[^ ^59^, ^60^, ^61^, ^62^, ^63^, ^64^ ^]^ In this study, as the administration period was extended, the reduction in the abundance of potentially harmful bacteria became more pronounced. This decline may be attributed to the synergistic effects of the notably elevated levels of sIgA, mucin, probiotics, and SCFA after SEC2 administration. This study indicates that long‐term oral administration of SEC2 induces a beneficial shift in the structural composition of the mouse gut microbiota toward intestinal health.

SCFAs are mainly produced by gut microbiota metabolism and serve as a vital link between the gut microbiota and intestinal mucosal immunity. SCFAs perform various functions, including providing energy to intestinal epithelial cells, maintaining the epithelial barrier function, stabilizing intestinal pH, regulating immune cell responses, and inhibiting tumor cell growth and intestinal inflammatory reactions.^[^ ^65^, ^66^, ^67^, ^68^ ^]^ SCFAs are involved in regulating the immune response by inducing T cell differentiation and the secretion of immune factors through G protein‐coupled receptors (GPCR).^[^ ^48^ ^]^ Additionally, SCFAs help maintain the integrity of the intestinal barrier by increasing the expression of ZO‐1 and Claudin‐1 and influencing the redistribution of Occludin.^[^ ^69^ ^]^ Reports indicate that SCFAs alter lipid metabolism and effectively enhance the stemness of intestinal stem cells, thereby promoting epithelial cell development.^[^ ^70^, ^71^ ^]^ Additionally, T and B cells, which play crucial roles in maintaining intestinal villus structure,^[^ ^72^ ^]^ proliferate in response to SEC2. Long‐term oral administration of SEC2 promotes intestinal villus growth and enhances intestinal absorption. Administration of SEC2 promotes the growth of intestinal epithelial cells and secretion of mucosal proteins, which provide a habitat for more functional microbiota enriched in the intestine. This promoted the production of SCFA. Acetic, propionic, butyric, and valeric acid levels substantially increased in SEC2‐treated mice, particularly on days 7 and 14. The increase in these major SCFAs corresponds to intestinal immune cell differentiation and tight junction protein expression. These findings indicate that oral administration of SEC2 directly engages GALT and activates the intestinal immune response. This interaction leads to alterations in gut microbiota, resulting in the production of SCFAs. These SCFAs, in turn, promote both intestinal immunity and barrier functions.

Due to the dissimilar sensitivities between humans and mice to SAg, the experimental results of this study should not be directly extrapolated to humans. Nonetheless, our study implies that oral administration of SEC2 at an appropriate dose has the potential to foster intestinal health and enhance systemic immunity.

## 4 Conclusion

In this study, orally administered SEC2 tolerated the digestive environment and reached the intestines and even the bloodstream in the form of intact protein molecules. Orally administered SEC2 activated cellular immunity mediated by CD4^+^ and CD8^+^ T cells, as well as humoral immunity mediated by CD19^+^ B cells and sIgA in PPs and MLNs. SEC2 alters the community structure of the gut microbiota and reinforces potential probiotics and their metabolite SCFA. Through these mechanisms, the oral administration of SEC2 enhances intestinal and peripheral immune functions and improves intestinal barrier function. This study provides valuable insights into the development of SEC2 as an orally administered immunostimulant medication and its potential applications in safe and effective immunotherapy.

## 5 Experimental Section

### Reagents and Antibodies

The expression vector containing SEC2 encoding gene was constructed and preserved in our laboratory. Recombinant SEC2 was expressed in *Escherichia coli* BL21 (DE3) strains and purified as previously described.^[^ ^7^ ^]^ The horseshoe crab reagent gel assay detected endotoxin levels in purified SEC2 below the detection range. The Alexa Fluor 647 fluorescent dye was purchased from Invitrogen (Carlsbad, CA, USA). Intact SEC2‐specific ELISA Kit was customized by Absea (Beijing, China). Anti‐mouse monoclonal antibodies CD3‐PE, CD4‐FITC, CD8α‐PerCP Cy5.5, CD19‐FITC were purchased from BD Pharmingen (New Jersey, USA). Anti‐mouse monoclonal antibodies CD45, IgA, Claudin‐1 were purchased from Proteintech Group (Wuhan, China). The ELISA kit for mouse IL‐2 was purchased from Dakewe (Shenzhen, China). RT‐qPCR primers for β‐actin, TNF‐α, INF‐γ, IL‐10, IL‐2, Muc2, Muc3, Claidin‐1, Occludin‐1, and ZO‐1 were purchased from Sangon Biotech (Shanghai, China). The ELISA kit for sIgA was purchased from Sangon Biotech (Shanghai, China). The RNAiso Plus Kit, Primescript RT Reagent Kit with gDNA eraser, and SYBR PreMix Ex Taq Kit were purchased from Takara Bio (Dalian, China). The chemical standards (acetic acid, propionic acid, butyric acid, valeric acid, isobutyric acid, and isovaleric acid) were purchased from Anpel (Shanghai, China). 2,3,4,5,6‐Pentafluorobenzyl bromide (PFBBr) was purchased from Aladdin (Shanghai, China).

### In Vivo Distribution of SEC2

The distribution of SEC2 in mice was investigated using In Vivo Imaging. SEC2 was fluorescently stained using Alexa Fluor 647 ester dye before being administered IG at a dose of 5 mg kg^−1^ in a 200 µL volume. The mice were scanned at predetermined time points (0.5, 1, 2, 4, 8, 12, and 24 h after administration) using an IVIS Spectrum Imaging System (PerkinElmer, Massachusetts, USA) with excitation and emission filters of 650 and 665 nm, respectively. Prior to imaging, mice were anesthetized with tribromoethanol (1.25%) at the dose of 20 µL g^−1^ body weight, and their abdominal hair was shaved.^[^ ^73^ ^]^

SEC2 accumulation in the stripped digestive tract tissues was also analyzed. At each predetermined time point after intragastric administration, as described above, the mice were sacrificed, and GI tissues (stomach, small intestine, cecum, and colon) were isolated and scanned using the IVIS Spectral Imaging System. After imaging, GI tissues were homogenized separately in PBS using a high‐throughput tissue grinder (SciEbtz, China), and the supernatant was obtained by centrifugation at 12 000 × *g* for 20 min. Blood from the mice was isolated and incubated at 37 °C for 30 min before being transferred to 4 °C for overnight incubation. Serum was obtained by centrifugation at 3000 × *g* for 15 min. The concentration of intact SEC2 in the supernatant or serum was determined using Sandwich ELISA to assess the distribution and structural integrity of SEC2 after gavage.

### Animals and Experimental Design

Female specific‐pathogen‐free (SPF) BALB/C mice (6–8 weeks old, weight at 20 ± 2 g) were purchased from Beijing Vital River Laboratory Animal Technology (Beijing, China). All mice were kept in independently ventilated cages under sterile conditions with 12 h day/night cycles and free access to autoclaved water and sterile food. After one week of acclimatization, all mice were randomly assigned to 12 groups (n = 6 in each group) (Table S2, Supporting Information). Mice received SEC2 (LD group: 5 mg kg^−1^, MD group: 10 mg kg^−1^, and HD group: 20 mg kg^−1^) or PBS at a volume of 0.2 mL via oral gavage every day for 7 days, 14 days, or 28 days. The mice in all groups were weighed daily. The day after each time point, mice were sacrificed by intraperitoneal administration of tribromoethanol (400 mg kg^−1^ body weight), and the blood, intestinal tissues, spleens, and feces were collected for subsequent analysis. All mouse experiments were approved by the Institutional Animal Care and Use Committee of Shenyang Medical College (Approval No. SYYXY2022032802) and every attempt was made to reduce animal suffering.

### Flow Cytometry Analysis

The PPs, MLNs, and spleens from mice were ground through a 200‐mesh nylon screen to obtain lymphocytes separately. The lymphocytes were gently washed twice with pre‐cooled PBS buffer and were stained for surface markers using specific antibodies for mouse CD4‐FITC, CD8α‐APC, CD3‐PerCP‐Cy5.5, and CD19‐FITC. After gentle mixing, the mixture was incubated in the dark at 4 °C for 30 min. Flow cytometry was performed using a BD Accuri C6 Plus instrument, and the data were analyzed using the FlowJo V10 software.

### Histopathological and Immunohistochemical Analysis

The small intestinal tissues of the mice were collected and cut into 1 cm segments for histological observation (n = 3 per group). The tissues were fixed in 4% paraformaldehyde solution for 24 h, dehydrated, and embedded in paraffin blocks. The specimens from each group were cut into 5‐µm thick sections. Sections were stained with H&E or PAS solution. H&E staining was performed for histomorphometric analysis and histological scoring. Morphological structural changes, villus height, and crypt depth of the small intestine were analyzed using CaseViewer 5.0 software, villus height was determined by the length between the crypt‐villus junction and the top of the villus. Crypt depth was measured as the length between the bottom of the crypts and the crypt‐villus junction. At least five intestinal villi and crypts were counted per section. The histological scoring of the intestines was performed using the scoring system described in Table S3 (Supporting Information). PAS staining was used to analyze the goblet cells. The number of goblet cells per villus or crypt was measured and counted using Image‐Pro Plus software 6.0, and at least five intestinal villi and crypts were counted per section. The profiles of leukocytes infiltrating the intestine, IgA in the lamina propria, and tight junctions of the intestinal epithelium were respectively determined by IHC analysis of CD45, IgA, and Claudin‐1. The sections were observed using a Laser Scanning Confocal Microscope (LSCM), and statistical analysis was conducted using CaseViewer 5.0 and Aipathwell software (Servicebio, China).

### RNA Extraction and Gene Transcription Analysis

Intestinal tissues were ground at low temperatures using a high‐throughput tissue grinder (SciEbtz, China). Total RNA was extracted from the intestinal homogenate using the RNAiso Plus Total RNA Extraction Kit following the manufacturer's instructions. The concentration and purity of the extracted RNA were evaluated using a Nanodrop 2000 spectrophotometer (Thermo Scientific, Wilmington, DE, USA) and all samples were adjusted to the same concentration using DEPC water. The cDNA was obtained using PrimeScript RT reagent Kit with gDNA Eraser according to the manufacturer's protocol. Then, the real‐time quantitative PCR was performed using the LightCycler 96 Real‐time PCR System (Roche Applied Science, Basel, Switzerland) with SYBR Premix Ex TaqII Kit. Data were analyzed using 2^−ΔΔCt^ method,^[^ ^74^ ^]^ and the 2^–ΔΔCt^ of genes in the control group was set as 1. The β‐actin was used as the housekeeping gene for normalization. The primer sequences used in this study are listed in Table S4 (Supporting Information).

### ELISA Assay for sIgA and IL‐2

sIgA in the intestinal tissue and IL‐2 in the serum were detected by ELISA. Intestinal tissues were homogenized in PBS and centrifuged at 3000 × *g* for 20 min to obtain the supernatant. And, the serum was collected from blood samples by centrifugation at 1500 × *g* and 4 °C for 15 min to obtain the supernatant. The levels of sIgA and IL‐2 were quantified using ELISA kits according to the manufacturer's protocol. Absorbance was measured at 450 nm using a FLUOstar Omega spectrophotometer (BMG, USA).

### Fecal DNA Extraction and 16S rRNA Gene Sequencing

Total DNA was extracted from mouse fecal samples using a FastDNA SPIN Kit (MP Bio, USA), and the concentration and purity of the extracted DNA were evaluated using a Nanodrop 2000 spectrophotometer (Thermo Scientific, Wilmington, DE, USA). Polymerase chain reaction (PCR) amplification using barcoded fusion primers 338F/806R targets the V3‐V4 region of the bacterial 16S rRNA gene.^[^ ^75^ ^]^ The amplicons were merged on an Illumina MiSeq PE300 platform (Illumina Inc., San Diego, CA, USA) following the standard protocols of Majorbio Bio‐Pharm Technology Co. Ltd. (Shanghai, China). Raw sequencing data were uploaded to the NCBI for Biotechnology Information database (Accession Number: PRJNA1082098) and analyzed using the MajorBio Cloud Platform (https://www.majorbio.com). Low‐quality sequences (quality scores < 20, length < 50 bp) were filtered and removed using Quantitative Insights into Microbial Ecology software (QIIME, version 1.9.1). Operational taxonomic units (OTUs) were assigned to the reads using UPARSE (version 7.1) at a 97% sequence similarity threshold.^[^ ^76^ ^]^ The taxonomic identity of all phylotypes was determined using the SILVA ribosomal RNA gene database (version 138) with a comparison threshold set at 70%.^[^ ^77^ ^]^

### Extraction and Analysis of SCFAs from Feces

SCFAs were isolated from the mouse fecal samples. First, 50 mg of mouse feces were ground into a powder in liquid nitrogen, mixed with 170 µL of deionized water, 30 µL of 0.5 M Na_2_HPO_4_ solution and 600 µL of PFBBr acetone solution. After reaction in a water bath at 60 °C for 40 min, 200 µL of hexane was added to each sample and centrifuged at 3000 × *g* for 15 min, and the upper hexane phase was collected for analysis. The concentrations of SCFAs (acetic acid, propionic acid, butyric acid, valeric acid, isobutyric acid, isovaleric acid) were analyzed using Gas Chromatography‐Mass Spectrometry (Trace1300‐ISQ7000, Thermo Fisher, USA) with a Thermo Scientific TR‐WAX MC‐GC Column (30 m, 0.25 mm ID, 0.25 µm). The concentrations were calculated using an external standard method.

### Statistical Analysis

All experiments were independently repeated at least three times. To ensure an accurate comparative analysis across different values, all data were normalized before statistical analysis and plotting. All values are expressed as mean ± SD with the control group set as 1. Statistical analyses were performed to compare each treatment group with the corresponding control group at the same administration time. Statistical significance was determined by unpaired Student's t‐test or one‐way analysis of variance (ANOVA), followed by a suitable post hoc test using SPSS 22.0, and GraphPad Prism Software (version 9.0). Differences with *p* < 0.05 considered to be statistically significant, *p < 0.05, **p < 0.01, and ***p < 0.001. “ns”, not significant.

## Conflict of Interest

The authors declare no conflict of interest.

## Author Contributions

W.G. and H.Z. contributed equally to this work. W.G. performed methodology, formal analysis, investigation, visualization, and wrote the original draft. H.Z. performed methodology, formal analysis, investigation, and visualization. Z.Z. performed methodology, investigation, and visualization. M.X. performed conceptualization, methodology, provided resources, supervised, project administration, and wrote, reviewed and edited the original draft. X.L. and Z.H. performed investigation and visualization. X.F. performed investigation, and provide resources. X.L., X.W., and C.Z. provide resources and project administration. All authors provide approval for publication of the content.

## Supporting information



Supporting Information

## Data Availability

The data that support the findings of this study are available from the corresponding author upon reasonable request.
