# Supplementary material for: Continuous Oral Administration of the Superantigen Staphylococcal Enterotoxin C2 Activates Intestinal Immunity and Modulates the Gut Microbiota in Mice
Source: Adv Sci (Weinh). 2024 Sep 9;11(41):2405039. doi: 10.1002/advs.202405039 (PMC11538665; doi:10.1002/advs.202405039)
Supplement: Supplementary file 1 — Supporting Information [file ADVS-11-2405039-s001.docx]

Supporting Information

**Continuous oral administration of the superantigen Staphylococcal Enterotoxin C2 activates intestinal immunity and modulates the gut microbiota in mice**

*Wu Gu, Huiwen Zhang, Zhichun Zhang, Mingkai Xu^*^, Xiang Li, Zhiyang Han, Xuanhe Fu, Xu Li, Xiujuan Wang, Chenggang Zhang*

**
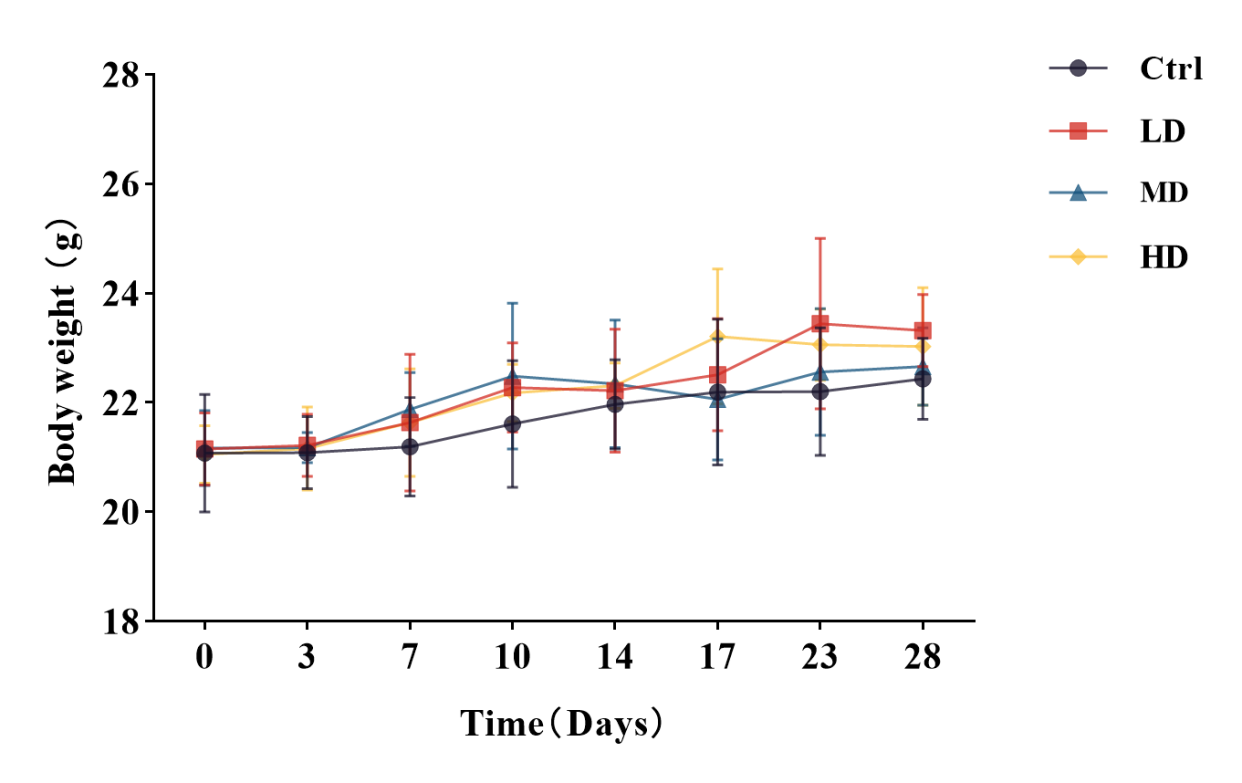
**

**Supplementary Figure 1.** Changes in the body weight of mice following continuous oral administration of SEC2. Data are presented as mean ± SD (n = 3).

**
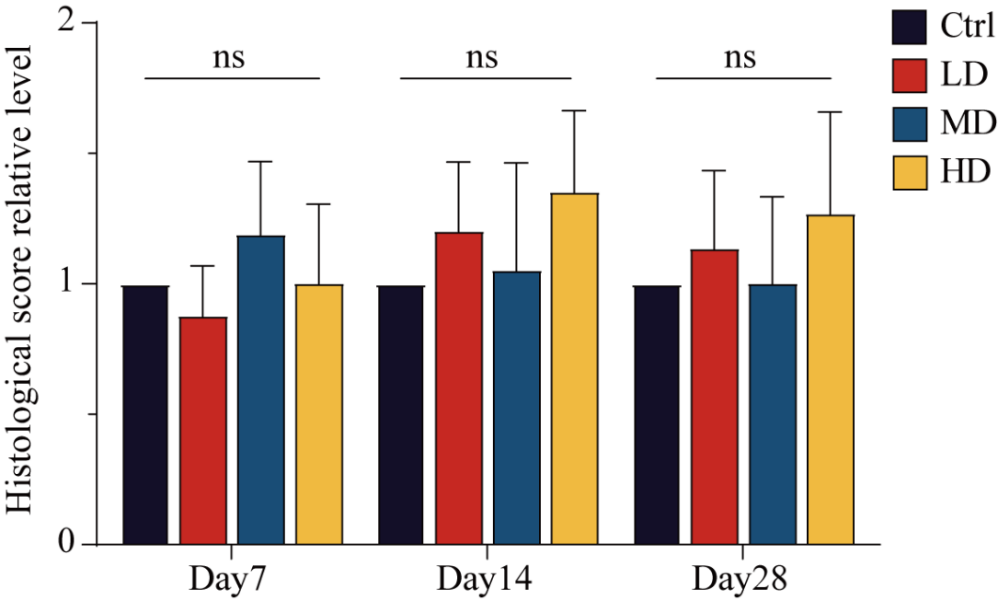
**

**Supplementary Figure 2.** Histological score of mice intestine after continuous oral administration of SEC2. Data were normalized with the control group set as 1 and presented as mean ± SD (n = 6). “ns”, not significant.


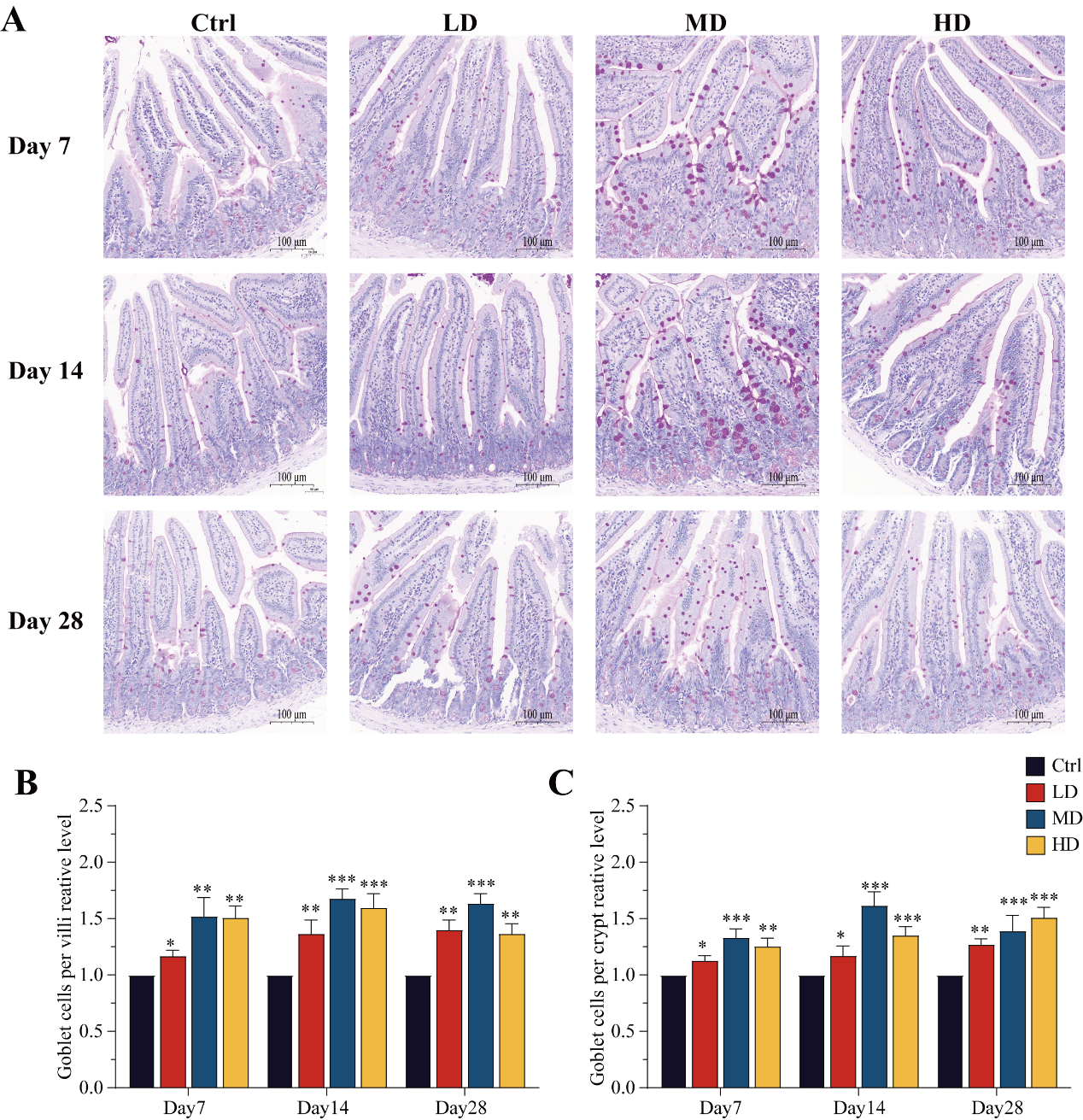


**Supplementary Figure 3.** Periodic acid-Schiff (PAS) staining of intestinal section. A) Goblet cells in mice intestine were evaluated by PAS staining. Scale bar = 100μm. Histograms of B) goblet cells per villi and C) goblet cells per crypt data presented through visual analysis. Data were normalized with the control group set as 1 and presented as mean ± SD, at least 5 villus or crypts per section were select for analyze. “*” represents significant differences between the treatment groups and their respective control groups. * *p* < 0.05, ** *p* < 0.01, *** *p* < 0.001.

**
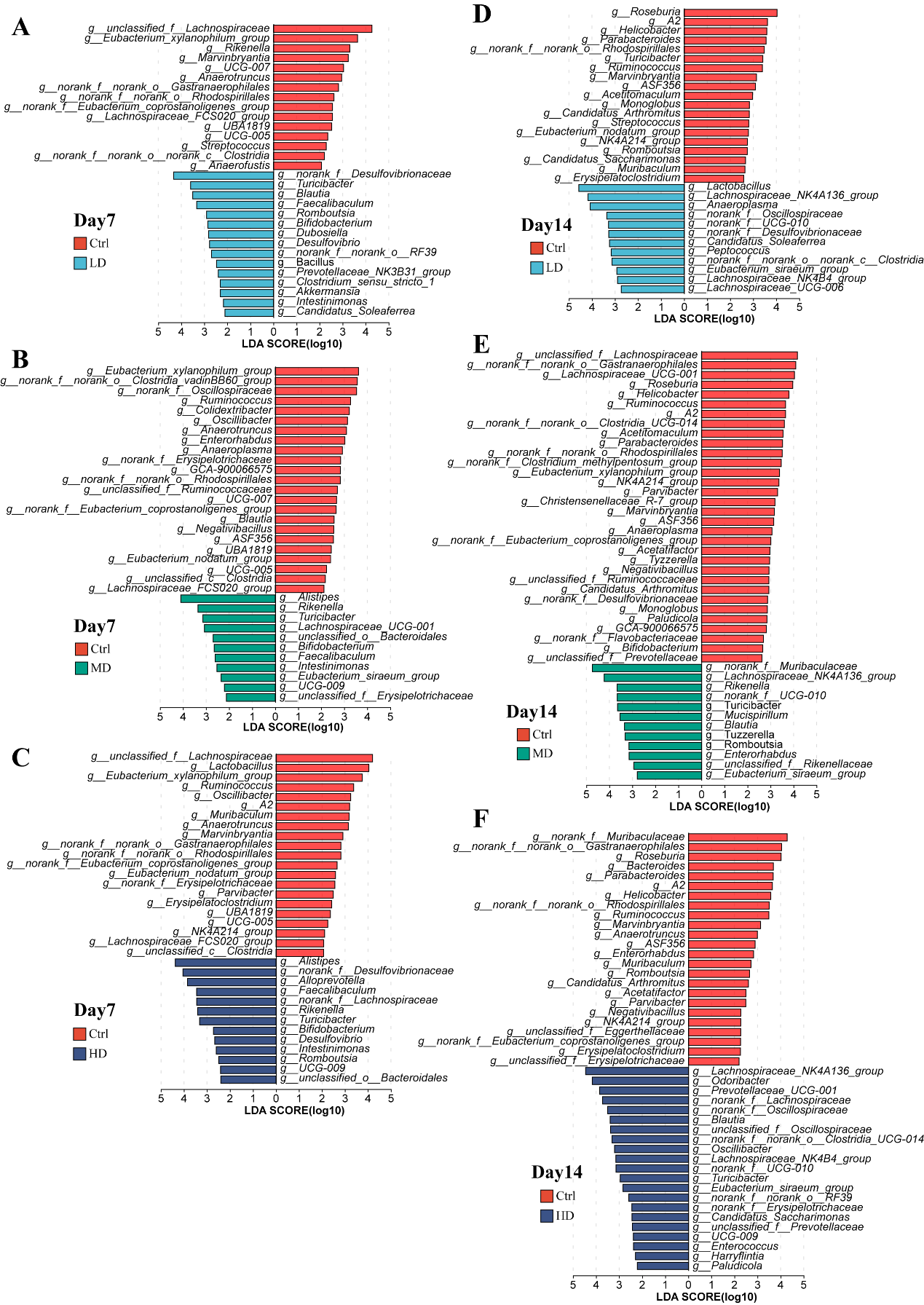
**

**Supplementary Figure 4.** LEfSe analyses in gut microbial communities. Histogram of Linear Discriminant Analysis (LDA) scores (LDA > 2.5) were calculated for features that differ in abundance at the genus level between treatment and control groups. A) - C) SEC2 treatment groups of LD, MD, and HD were administered continuously for 7 days. D) - F) SEC2 treatment groups of LD, MD, and HD were administered continuously for 14 days.


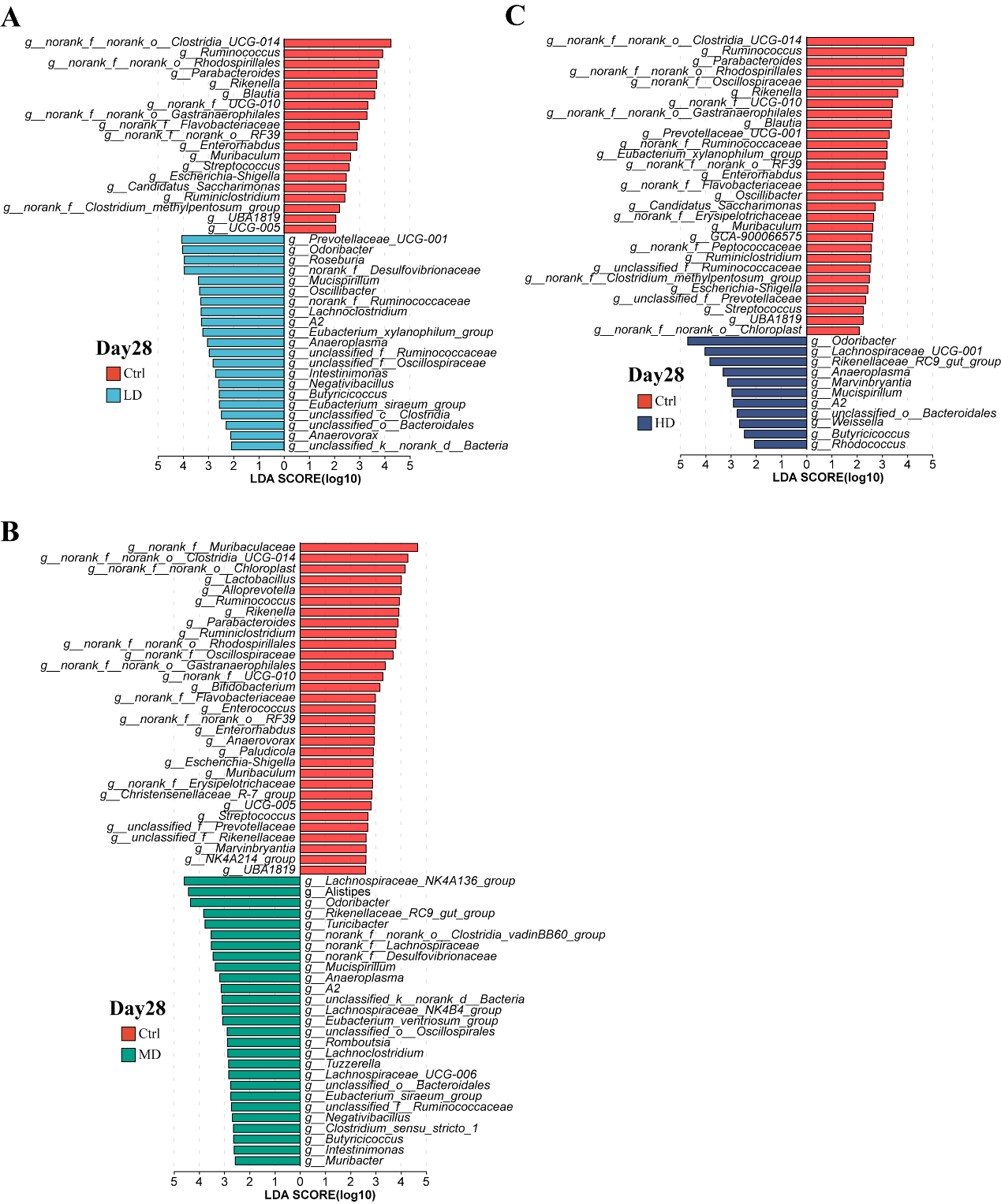


**Supplementary Figure 5.** LEfSe analyses in gut microbial communities. Histogram of Linear Discriminant Analysis (LDA) scores (LDA > 2.5) were calculated for features that differ in abundance at the genus level between treatment and control groups. A) - C) SEC2 treatment groups of LD, MD, and HD were administered continuously for 28 days.

**
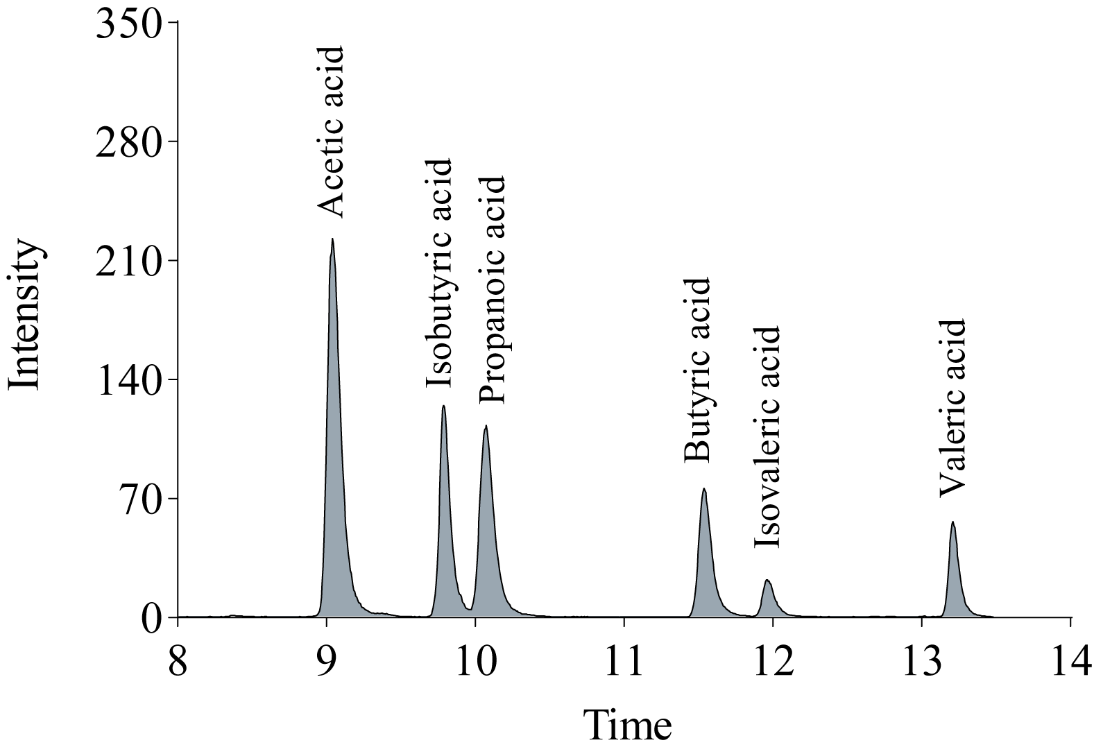
**

**Supplementary Figure 6.** Total ion current chromatogram of SCFAs

**Supplementary Table 1.** Standard curves of SCFAs

| SCFAs | Standard curve | R-squared (R^2^) |
| --- | --- | --- |
| Acetic acid | Y = 709467+7367.19*X | 0.9951 |
| Isobutyric acid | Y = -37175.9+5823.37*X | 0.9937 |
| Propanoic acid | Y = 5050.01+9345.52*X | 0.9941 |
| Butyric acid | Y = -15763.6+2062.4*X | 0.9928 |
| Isovaleric acid | Y = -8722.75+672.379*X | 0.9940 |
| Valeric acid | Y = -10271.7+606.877*X | 0.9927 |

**Supplementary Table 2.** Grouping of experiments.

| Duration  Dose  Group | 7 days | 14 days | 28 days |
| --- | --- | --- | --- |
| LD group | 5 mg·kg^−1^ SEC2 | 5 mg·kg^−1^ SEC2 | 5 mg·kg^−1^ SEC2 |
| MD group | 10 mg·kg^−1^ SEC2 | 10 mg·kg^−1^ SEC2 | 10 mg·kg^−1^ SEC2 |
| HD group | 20 mg·kg^−1^ SEC2 | 20 mg·kg^−1^ SEC2 | 20 mg·kg^−1^ SEC2 |
| Ctrl group | PBS | PBS | PBS |

**Supplementary Table 3.** Histological intestinal inflammation scoring system

| Feature score | Score | Description |
| --- | --- | --- |
| Inflammation severity | 0 | None |
|  | 1 | Mild |
|  | 2 | Moderate |
|  | 3 | Severe |
| Inflammation extent | 0 | None |
|  | 1 | Mucosa |
|  | 2 | Submucosa |
|  | 3 | Transmural |
| Villus and crypt damage | 0 | None |
|  | 1 | Basal 1/3 damage |
|  | 2 | Basal 2/3 damage |
|  | 3 | Loss of either villus or crypts |
|  | 4 | Both the villus and crypts were lost |
| Per cent involvement | 0 | 0% |
|  | 1 | 1-25% |
|  | 2 | 26-50% |
|  | 3 | 51-75% |
|  | 4 | 76-100% |

Histological criteria were used to evaluate the severity of inflammation. The score ranges from 0 to 14 (total score), which represents the sum of scores from 0 to 4 for the severity and extent of inflammation, villus and crypt damage, and the percentage of the intestine involved. All evaluations were conducted by observers who were unaware of the treatment groups.

**Supplementary Table 4. Sequences for RT-qPCR primers**.

| Primer name | Forward Primer（5’ to 3’） | Reverse Primer（5’ to 3’） |
| --- | --- | --- |
| β-actin | CTTCGCGGGCGACGAT | GACCCATTCCCACCATCACA |
| IL-2 | ATGAACTTGGACCTCTGCGG | GTCCACCACAGTTGCTGACT |
| IL-10 | GCATGGCCCAGAAATCAAGG | GAGAAATCGATGACAGCGCC |
| IFN-γ | AAGACAATCAGGCCATCAGC | CTGGACCTGTGGGTTGTTGA |
| TNF-α | CTGTAGCCCACGTCGTAGC | TTGAGATCCATGCCGTTG |
| Claudin-1 | GGGGACAACATCGTGACCG | AGGAGTCGAAGACTTTGCACT |
| Occludin-1 | TTGAAAGTCCACCTCCTTACAGA | CCGGATAAAAAGAGTACGCTGG |
| ZO-1 | GCCGCTAAGAGCACAGCAA | TCCCCACTCTGAAAATGAGGA |
| Muc 2 | ATGCCCACCTCCTCAAAGAC | GTAGTTTCCGTTGGAACAGTGAA |
| Muc 3 | GCCGTGAATTGTATGAACGGA | CGCAGTTGACCACGTTGACTA |
